# Metal Oxide Nanoparticles Containing Clotrimazole to Suppress Photodegradation of Poly(Vinyl Chloride) Thin Films

**DOI:** 10.3390/polym15071632

**Published:** 2023-03-24

**Authors:** Noor Emad, Gamal A. El-Hiti, Emad Yousif, Benson M. Kariuki

**Affiliations:** 1 Department of Chemistry, College of Science, Al-Nahrain University, Baghdad 64021, Iraq; st.noor.emadf@ced.nahrainuniv.edu.iq (N.E.); emad.yousif@nahrainuniv.edu.iq (E.Y.); 2 Department of Optometry, College of Applied Medical Sciences, King Saud University, Riyadh 11433, Saudi Arabia; 3 School of Chemistry, Cardiff University, Main Building, Park Place, Cardiff CF10 3AT, UK; kariukib@cardiff.ac.uk

**Keywords:** poly(vinyl chloride), ultraviolet irradiation, photodegradation, metal oxide nanoparticles, polymer weight loss, clotrimazole

## Abstract

Pol(vinyl chloride) or PVC has functional properties that enable its use in many industrial applications. It suffers from aging, however, in harsh conditions (e.g., elevated temperature or high humidity levels) if oxygen is present. One way to enhance the photostability of PVC is to blend it with additives. Thus, thin films were made by mixing PVC with clotrimazole, and five metal oxide (titanium, copper, cobalt, chromium, and nickel oxides) additives. The metal oxides and clotrimazole were added at concentrations of 0.1 and 0.5% by weight, respectively. The effect of the metal oxide nanoparticles accompanied by clotrimazole on the photodegradation of PVC was then assessed. The results indicated that the additives have a stabilizing effect and protect PVC against photodegradation significantly. The formation of polymeric fragments of small molecular weight containing carbon-carbon double bonds and carbonyl groups was lower in the blends containing metal oxide nanoparticles and clotrimazole than in unblended PVC. Similarly, the decrease in weight was much less for the films blended with additives. Additionally, surface analysis of the irradiated polymeric films showed significantly lower damage in the materials containing additives. The most effective additive in the stabilization of PVC was nickel oxide nanoparticles. The metal oxides are highly alkaline and act as scavengers for the hydrogen chloride produced during the photodegradation of PVC. They additionally act as peroxide decomposers. In contrast, clotrimazole can absorb harmful radiation and act as an ultraviolet absorber due to its heteroatom and aromatic content. Thus, the use of a combination of metal oxide nanoparticles and clotrimazole led to significant improvement in the resistance of PVC toward photodegradation.

## 1. Introduction

Plastics are ubiquitous synthetic polymers that are produced in huge quantities to meet demand. They have many applications that require a variety of shapes and forms. Plastics can be used to produce, for instance, components for building construction, cars, aircraft, medical devices, and packing. The demand for plastics has increased by more than 20-fold in the last 50 years [1,2]. Poly(vinyl chloride), PVC, is one of the common synthetic plastics involved in many applications [3]. PVC has chemical and physical properties that make it amenable to a wide range of applications. In addition, PVC is affordable, easy to be molded and color, and can be produced in a variety of shapes with a range in hardness [4]. PVC can be used in electrical and sound insulation, building materials, household goods, medicinal devices, electronics, cables, and automotive components [5,6,7,8]. Additionally, PVC contains a high proportion of chlorine and therefore can be employed as a fire retardant. A downside of PVC, however, is photodegradation at high temperatures in an oxygenated atmosphere. Accordingly, there is a need for the development of new materials to be incorporated into PVC in order to increase durability in addition to the maximization of performance, lowering of production costs, and reduction in detrimental environmental impact [9,10,11].

PVC oxidative degradation occurs when the polymer is exposed to ultraviolet (UV) radiation (e.g., sunlight) in an environment containing an appreciable amount of oxygen [12,13,14,15,16]. Photodegradation involves the formation of radicals following the absorption of UV radiation. PVC is not expected to absorb light with a wavelength beyond 200 nm. However, PVC contains impurities from the manufacturing process (e.g., carbonyl and peroxide-containing compounds) at a low concentration in defect sites at which the photodegradation process starts [17]. PVC photodegradation causes chain scission, cross-linking, and branching leading to the formation of reactive fragments incorporating carbonyl (C=O) and alkene (C=C) groups. In addition, dehydrochlorination takes place with the elimination of hydrogen chloride (HCl) and other volatiles [18]. The effect on the material is discoloration, weight loss, cracks, and dark spots [18]. The addition of stabilizers to PVC during manufacture can reduce the harmful effects of harsh conditions (e.g., sunlight and high temperature) by increasing its photostability and thereby extending its useful lifetime [19,20,21,22].

For the additives to be effective against the photodegradation of PVC, they need to fulfill certain criteria. The additives should be stable, non-volatile, non-toxic, and active at a low concentration, and not lead to a color change. They should also contain heteroatoms and aromatic and/or heterocyclic moieties, as well as being easy and inexpensive to synthesize. As a consequence of their composition, the additives should have the ability to absorb harmful radiation, scavenge HCl and peroxides, and decompose reactive species (e.g., free radicals containing species) [23,24,25]. Various additives have been used on a commercial scale to increase PVC photostability [26,27,28,29,30]. The most common PVC commercial additives include metals, metal oxides, benzophenones, benzotriazoles, phthalates, *tris*(di-*tert*-butylphenyl)phosphite, and tetrachlorobiphenyl. These additives commonly act as UV absorbers and HCl quenchers. However, many of these PVC stabilizers pose a danger to human and animal populations and the environment, and therefore their use has been banned [31,32,33]. In recent years, attention has shifted to the synthesis of new additives (e.g., aromatics, heterocycles, Schiff bases, and organometallics) for application against PVC photodegradation [34,35,36,37]. It should be noted that most PVC photostabilizers contain aromatic moieties that are capable of absorbing UV irradiation directly [34].

Nanoparticles (NPs) have become the focus of researchers in various disparate fields due to their superior physical, chemical, and biological properties. Generally, NPs are synthesized using one of two approaches known as top-down and bottom-up processes. The top-down approach requires a mechanical process in which bulk material is crushed or break down into fragmented NPs. Alternatively, the bottom-up method involves synthesis of NPs through chemical processes [38,39]. NPs have different physical characteristics (e.g., porosity, surface area, size, and geometry) and can therefore be utilized in different applications (e.g., as antimicrobials, modifiers, membranes, nanofillers, and additives) [40,41,42]. They are becoming particularly important in healthcare and biomedical applications as they offer solutions to many challenges associated with many current materials used in the medicinal field. As an example, silver NPs have been found to have antioxidant and antimicrobial activities [43,44]. Recently, metal oxide NPs have been tested as photostabilizers for PVC [45,46]. The results indicated that the damage in the PVC chains due to UV photoirradiation was reduced by the presence of the metal NPs.

Recently, several groups of organometallics have been synthesized and used as PVC photostabilizers [47,48,49,50]. The current research involves the assessment of a combination of several metal oxide NPs and clotrimazole as PVC photostabilizers with the advantage that no additional synthetic procedures are involved to produce the additives. Metal oxides can act as bases to scavenge HCl eliminated from PVC chains due to photodegradation. On the other hand, clotrimazole is an excellent candidate to be part of a PVC additive. It is a highly stable solid, aromatic, and contains a high proportion of heteroatoms. Therefore, clotrimazole is expected to act as a UV absorber.

## 2. Materials and Methods

### 2.1. Materials and Instruments

PVC (molecular weight = ca. 233,000; degree of polymerization = ca. 800) was sourced from Petkim Petrokimya (Istanbul, Turkey). Clotrimazole (98%), metal oxide NPs (98–99%), and analytical grade solvents were obtained from Merck (Gillingham, UK). The diameters of titanium oxide (TiO_2_), copper oxide (CuO), cobalt oxide (Co_3_O_4_), chromium oxide (Cr_2_O_3_), and nickel oxide (NiO) were 15, 56, 15,50, and 31 nm, respectively. IR spectra were recorded on an FTIR Shimadzu 8400 spectrophotometer (Tokyo, Japan). The films were irradiated using UV light (λ_max_ = 313 nm; light intensity = 6.2 × 10^−9^ Einstein dm^−3^ × s^−1^) at 25 °C using an accelerated weather tester (Q-Panel Company; Homestead, FL, USA). The tester has two fluorescent lamps (40 watts) on the sides and the films were placed at a distance of 10 cm from the sources and oriented parallel to the lamps. To ensure uniform irradiation, the polymeric materials were rotated regularly. Optical images of the surface of PVC films were recorded on a Meiji Techno microscope (Tokyo, Japan). A SIGMA 500 VP microscope (Carl Zeiss Microscopy; White Plains, NY, USA) was used to record the scanning electron microscopy (SEM) images and the energy dispersive X-ray (EDX) maps of the films. A Veeco instrument (Plainview, NY, USA) was used to obtain the atomic force microscopy (AFM) images.

### 2.2. Preparation of PVC Films

#### 2.2.1. Preparation of Blank PVC Films

The unblended PVC film was prepared using the solvent casting method. PVC (5 g) was first stirred in tetrahydrofuran (THF) as a solvent (100 mL) at 25 °C for 3 h. The mixture was then sonicated for 1 h to remove air bubbles and transferred into a clean glass plate containing 15 holes with a thickness of ca. 40 μm. The plate was left for a day for the THF to evaporate. The PVC films obtained were dried in a vacuum oven at 40 °C for 8 h to ensure the complete elimination of solvent residue.

#### 2.2.2. Preparation of Modified PVC Films

**Figure 1:**
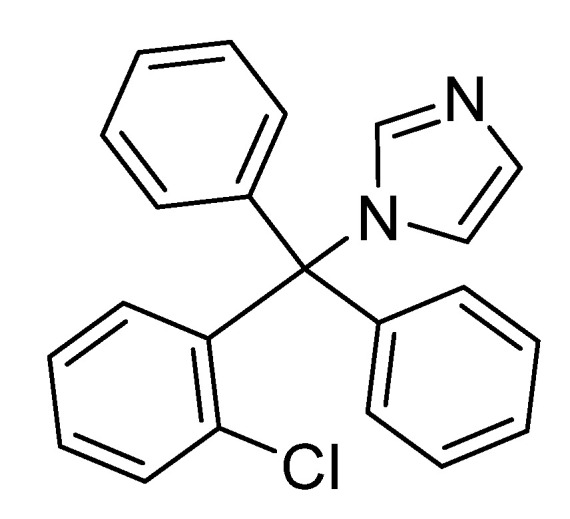
Structure of clotrimazole which is represented by L in the discussion.

The procedure was similar to that described in Section 2.2.1. In addition to the PVC (5 g), clotrimazole (Figure 1; 0.5% by weight, i.e., 25 mg) and appropriate metal oxide NPs (TiO_2_, CuO, Co_3_O_4_, Cr_2_O_3_, and NiO; 0.1% by weight, i.e., 5 mg) were added. Since small quantities (1% by weight) of metal oxide NPs were used, no color changes were observed in the resulting PVC films.

### 2.3. UV Radiation of PVC Films

The PVC films were kept under UV light (λ_max_ = 313 nm; light intensity = 6.2 × 10^−9^ Einstein dm^−3^ × s^−1^) for different periods in the range 50–300 h (i.e., 50, 100, 150, 200, 250, and 300 h) at 25 °C.

## 3. Results and Discussion

### 3.1. Assessment of PVC Photodegradation by FTIR Spectrophotometry

The unblended PVC film and the blends with clotrimazole (L) and metal oxide NPs were irradiated for 50–300 h with samples being analyzed every 50 h to determine the degree of photodegradation. IR spectroscopy is a useful method for the assessment of the effect of additives on photodegradation. Irradiation of PVC results in bond breaking, the elimination of HCl, and the formation of small fragments containing alcohol (OH), alkene (C=C), and ketone (C=O) groups [51,52].

The FTIR spectrum of the irradiated PVC showed a broad absorption band corresponding to the OH that appeared within the region of 3500 cm^−1^. It shows strong absorption bands that appeared at 1730 and 1615 cm^−1^, corresponding to the stretching vibrations of the C=O and C=C, respectively. Additionally, the FTIIR spectrum of PVC shows the presence of a peak that appeared at 1328 cm^−1^ corresponding to the bending vibrations of the C–H (from the CHCl residues of the PVC polymeric chains). Moreover, the spectrum shows bands corresponding to the C–C, C–Cl, and C–H (from the CH_2_ group) groups. The intensity increase in peaks corresponding to the C=O (1730 cm^−1^) and C=C (1615 cm^−1^) groups was assessed and compared to a reference peak. The C–H bond (1328 cm^−1^) of CH_2_ groups in the polymeric chain was used for comparison since irradiation has minimal effect on its intensity. The frequency corresponding to the C=O and C=C absorption bands is consistent with those reported [47,48,49,50]. However, the position of the C=O absorption band may vary depending on the type of carbonyl-containing fragments produced (aliphatic ketone, acid chloride, or chloroketone). As an example, the absorption bands for the C=O group of dichloroketone and acid chloride appear at 1745 and 1785 cm^−1^, respectively [53].

**Figure 2:**
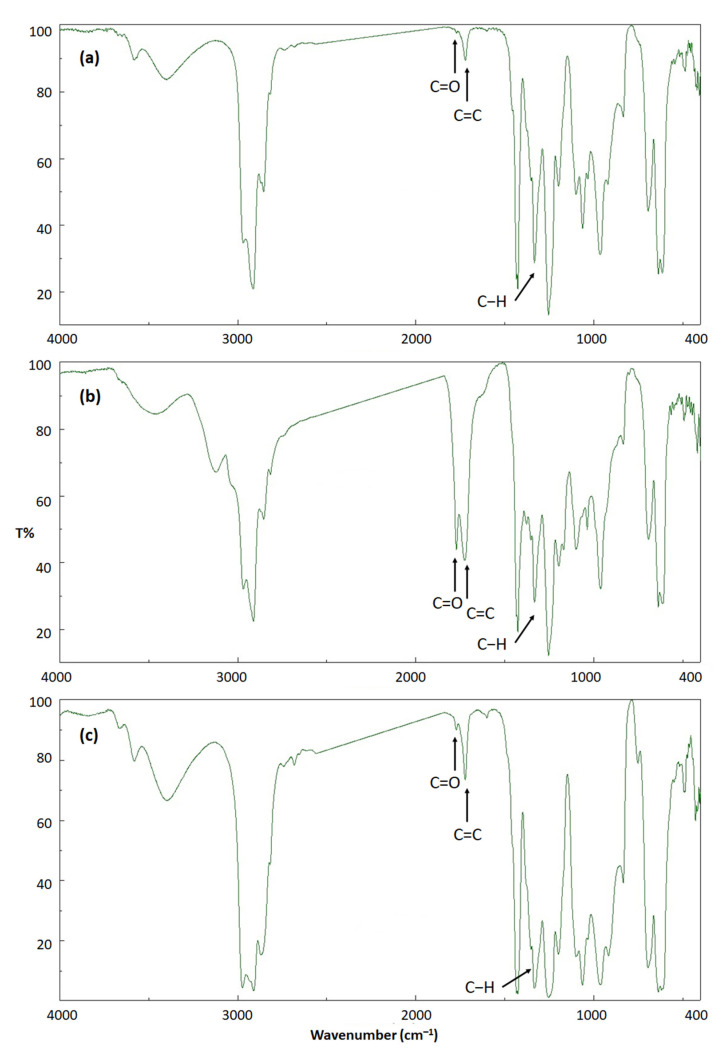
IR spectra of (**a**): unblended PVC before irradiation, (**b**): unblended PVC after 300 h of irradiation, and (**c**): PVC/clotrimazole/Ni NPs blend after 300 h of irradiation.

The IR spectra for the non-irradiated and irradiated (300 h) unblended PVC films and the irradiated blend containing both clotrimazole and Ni NPs are shown in Figure 2. The spectra clearly indicated the presence of fragments containing both the C=C and C=O groups after irradiation of PVC. However, the intensity of the absorption bands for these groups is lower in the case of the PVC blend containing both clotrimazole and Ni NPs compared to the pure film. The efficiency of the additives as PVC photostabilizers are due to the combined effect of both metal oxide NPs and ligand. The effectiveness of metal oxide NPs as PVC stabilizers could be due to their basicity thus acting as HCl scavengers. The strength of metal acidity is highly dependent on the ratio of charge to size, electronegativity, and hardness [54]. However, the basicity of metal oxides is not the only factor that contributes to their ability to act as PVC stabilizers. The diameter, size, geometry, and molecular weight of metal oxide NPs could play a role in their effectiveness as additives for PVC. In principle, metal oxide NPs can act as PVC additives on their own. However, this hypothesis needs to be tested.

**Figure 3:**
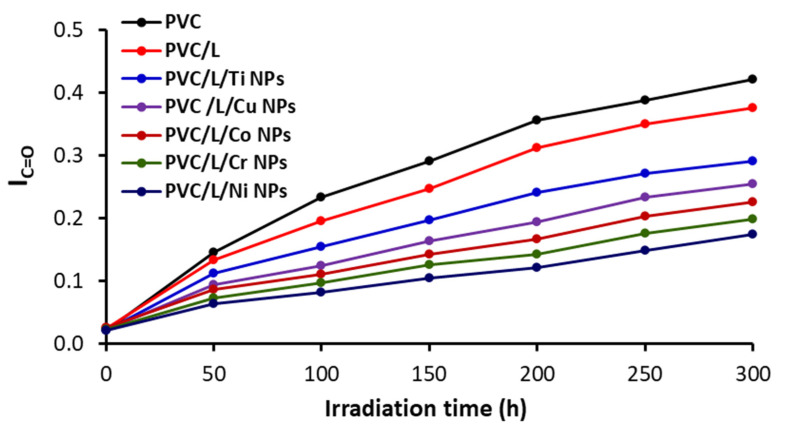
The I_C=O_ index as a function of irradiation time for PVC films.

**Figure 4:**
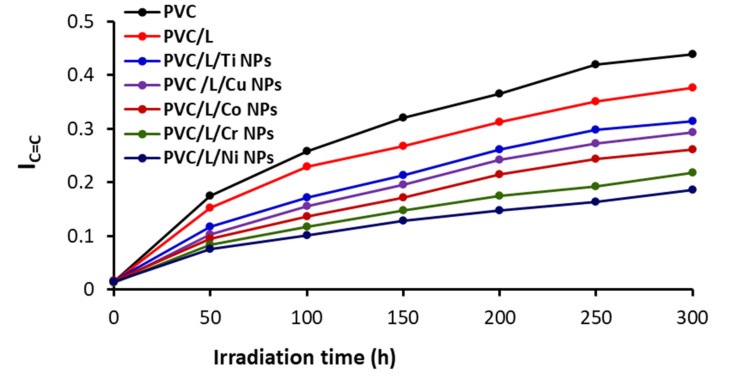
The I_C=C_ index as a function of irradiation time for PVC films.

After every 50 h period of irradiation, the absorbance for C=O or C=C (As) and that for the reference peak (Ar; C–H bond of the CH_2_ group) were determined. The indexes (Is) for both functional groups (IC=O and IC=C) were calculated using Equations (1) and (2) based on both the transmittance (T) and absorbance (A) [55]. Figure 3 and Figure 4 show the changes in both the I_c=o_ and I_c=c_ during irradiation, respectively.

(1) A=2−log T%

(2) Is=As/Ar

The I_c=o_ and I_C=C_ were highest for the unblended PVC film and increased as irradiation progressed. All additives resulted in a noticeable reduction in the I_C=O_ and I_C=C_. The clotrimazole (L) lead to some improvement in PVC photostability but much less than in the cases where NPs were used. The I_c=o_ after 300 h of irradiation were 0.42, 0.38, 0.29, 0.25, 0.23, and 0.17 for the films containing pure PVC, PVC/L, PVC/L/Ti NPs, PVC/L/Cu NPs, PVC/L/Co NPs, PVC/L/Cr NPs, and PVC/L/Ni NPs, respectively. Similarly, the I_C=C_ were PVC (0.44), PVC/L (0.38), PVC/L/Ti NPs (0.31), PVC/L/Cu NPs (0.29), PVC/L/Co NPs (0.26), PVC/L/Cr NPs (0.22), and PVC/L/Ni NPs (0.19) at the end of the process. The greater decrease in both I_c=o_ and I_C=C_ for the films blended with metal oxide NPs is clear and indicates the capabilities of such additives to increase PVC photostability. The Ni NPs led to the most noticeable reduction in PVC photodegradation. The effectiveness of NPS in stabilizing PVC decreases in the order Ni, Cr, Co, Cu, and Ti. A similar observation was made when a combination of Ni NPs and captopril as a PVC additive was compared with Cu and Co (i.e., Ni > Co > Cu NPS) [46].

### 3.2. Assessment of PVC Photodegradation by Weight Loss

**Figure 5:**
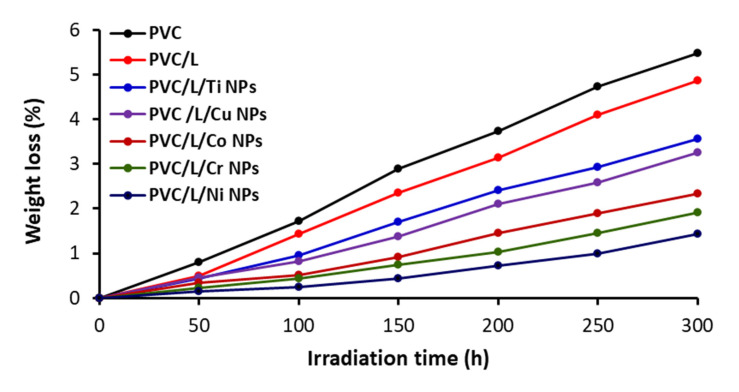
The weight loss percentage as a function of PVC film UV irradiation time.

Oxidative degradation of PVC can lead to undesirable effects such as color changes, elimination of volatiles (e.g., HCl), and formation of small fragments. A loss in polymer weight can also accompany the process [56]. The weight loss percentage is directly proportional to the amount of PVC photodegradation and the duration of irradiation. Thus, the films were weighed pre-irradiation (W0), irradiated with a UV light, and then reweighed (Wt) at 50 h intervals up to 300 h of irradiation. Equation (3) was used to calculate the percentage of PVC weight loss [57]. The results are shown in Figure 5.

(3) $$\mathrm{Weight}\;\mathrm{loss}\;\left(\%\right)=\frac{W0-\mathrm{Wt}}{W0}\times 100\;$$

The highest weight loss occurred when no additives were used. Clotrimazole on its own showed some degree of PVC protection, but combinations of clotrimazole and metal oxide NPs were more efficient as PVC additives. The film containing Ni NPs showed the lowest loss in weight in agreement with the results from the analysis of the IR spectroscopy functional group index (Figure 3 and Figure 4). For example, the weight losses (%) post-irradiation (i.e., after irradiation for 300 h) were 5.5, 4.9, 3.3, 2.3, 1.9, and 1.4 for the films containing pure PVC, PVC/L, PVC/L/Ti NPs, PVC/L/Cu NPs, PVC/L/Co NPs, PVC/L/Cr NPs, and PVC/L/Ni NPs, respectively.

### 3.3. Assessment of PVC Photodegradation by Surface Analysis

**Figure 6:**
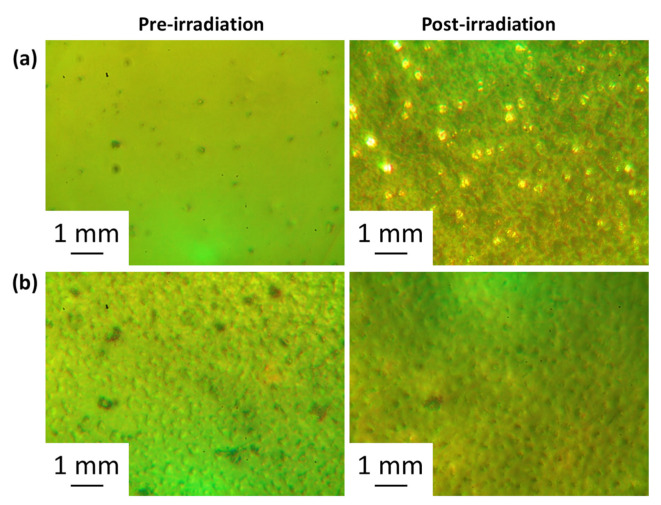
Pre- and post-irradiation (300 h) microscopy images of the films of (**a**): pure PVC and (**b**): PVC containing clotrimazole.

**Figure 7:**
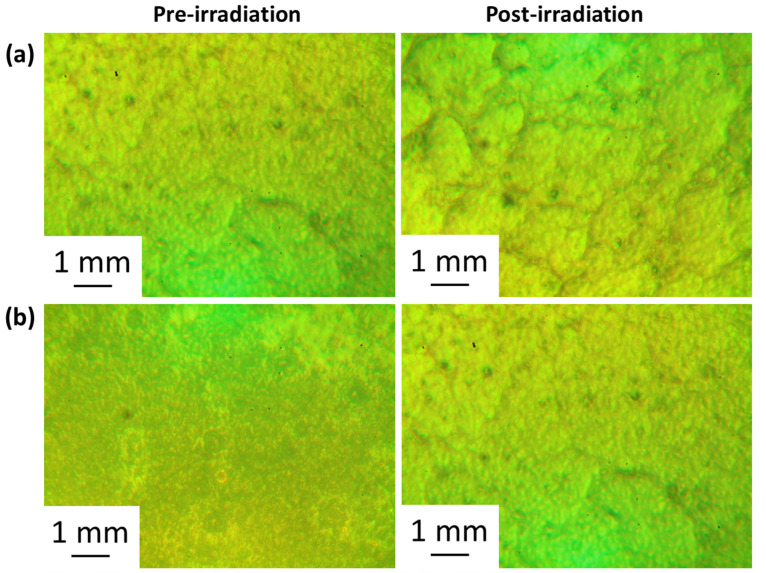

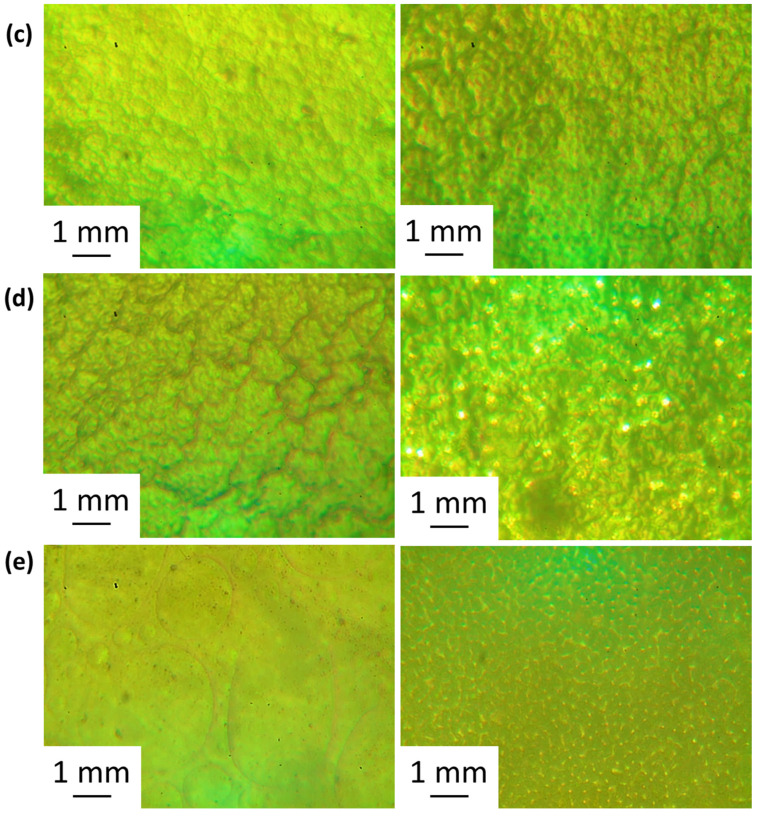
Pre- and post-irradiation (300 h) microscopy images of the films of (**a**): PVC/L/Ti NPs, (**b**): PVC/L/Cu NPs, (**c**): PVC/L/Co NPs, (**d**): PVC/L/Cr NPs, and (**e**): PVC/L/Ni NPs.

The inspection of the surface of PVC films can provide information on the damage caused by photodegradation. Irradiation can cause decomposition and chain scission of PVC leading to spotty, heterogenous, and rough surfaces. Different microscopes were used to assess for irregularities, deformations, roughness, graininess, darkness, spottiness, and crakes in the surface of the irradiated PVC film [58,59,60]. Irradiation led to a change in color, cracking, and dark spots whereas the surfaces of the non-irradiated polymers were smooth and regular. The optical microscopy images of the irradiated and nonirradiated PVC films are shown in Figure 6 and Figure 7. For example, the addition of clotrimazole reduces the homogeneity of the non-irradiated PVC film (Figure 6). However, following irradiation, the homogeneity of the surface of the irradiated PVC blend containing clotrimazole has not changed and was much better than that of the pure PVC film. This is clear evidence that the ligand itself can act as a PVC photostabilizers on its own. The images show noticeable surface damage on irradiation when no clotrimazole and/or metal oxide NPs are present. The use of both clotrimazole and metal oxide NPs led to a reduction in PVC photodegradation indicating that they act as efficient stabilizers. The least damage was observed for the blends containing Ni NPs, in consistence with the results obtained from the assessment of IR spectra and weight loss.

**Figure 8:**
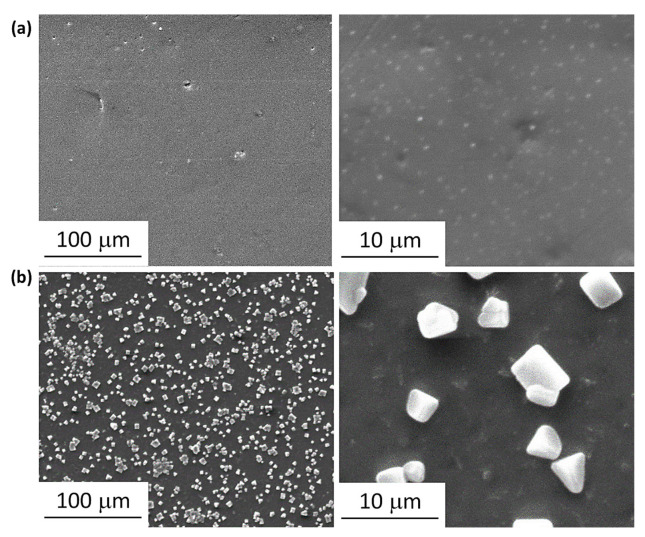
SEM Images of films of (**a**): unblended PVC and (**b**): PVC containing clotrimazole after irradiation.

**Figure 9:**
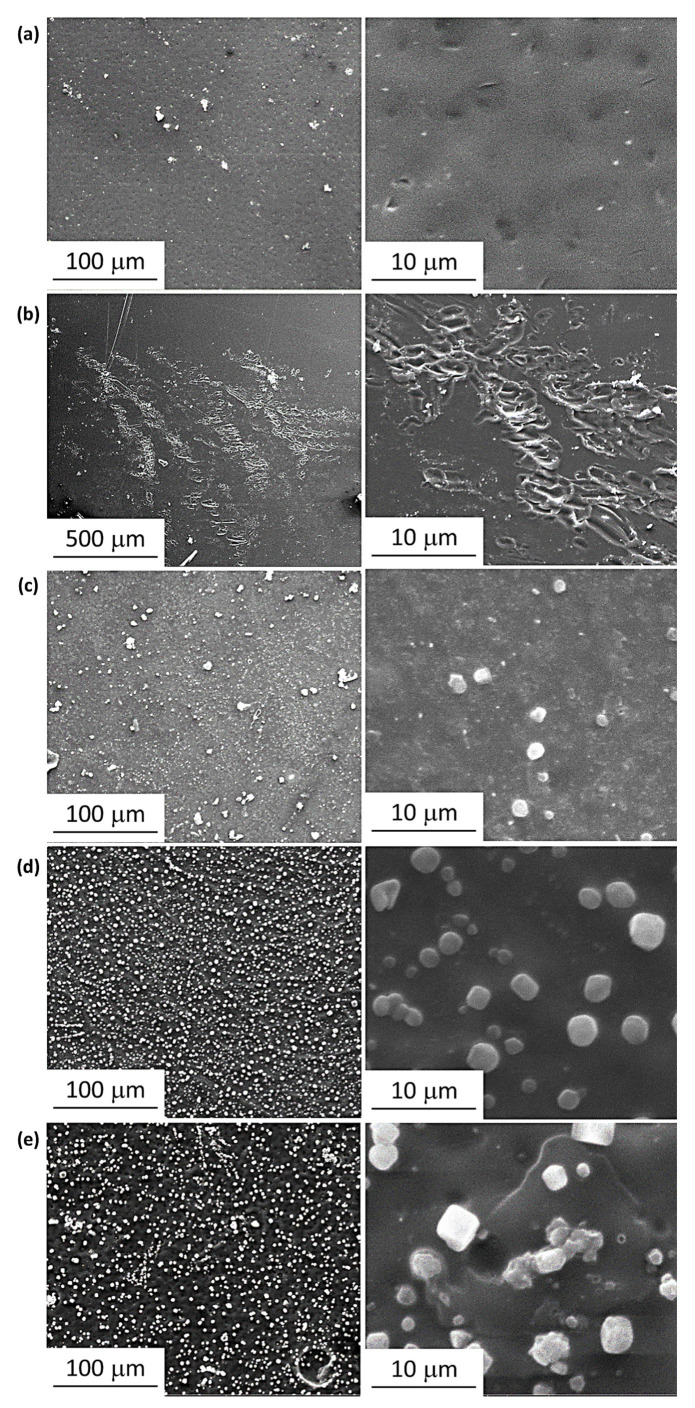
SEM Images of (**a**): PVC/L/Ti NPs, (**b**): PVC/L/Cu NPs, (**c**): PVC/L/Co NPs, (**d**): PVC/L/Cr NPs, and (**e**): PVC/L/Ni NPs after irradiation (300 h).

High-resolution SEM images of the PVC surfaces were also recorded. SEM images tend to be non-distorted and enable a clear assessment of surface irregularity and homogeneity [61]. In addition, the images provide information about particle size and shape as well as the diameters of pores and grooves and their distribution on the surface. The SEM images recorded for the PVC films after irradiation are shown in Figure 8 and Figure 9. The high rate of elimination of HCl and volatiles from the chains of PVC during irradiation forms grooves of varying shapes and sizes [62]. The SEM images show that the surfaces of the PVC blended with Cr and Ni NPs are similar in terms of pores’ shape, size, and distribution.

**Figure 10:**
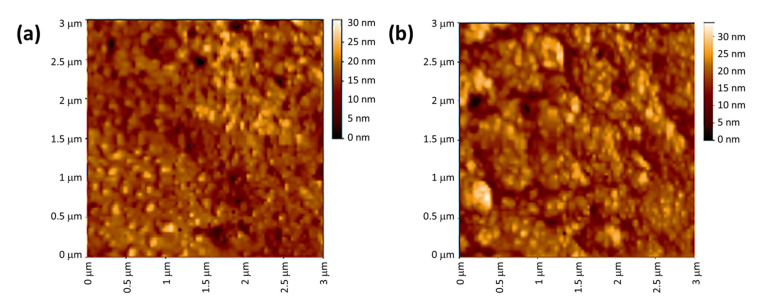
AFM Images of (**a**): pure PVC and (**b**): PVC containing clotrimazole after irradiation.

**Figure 11:**
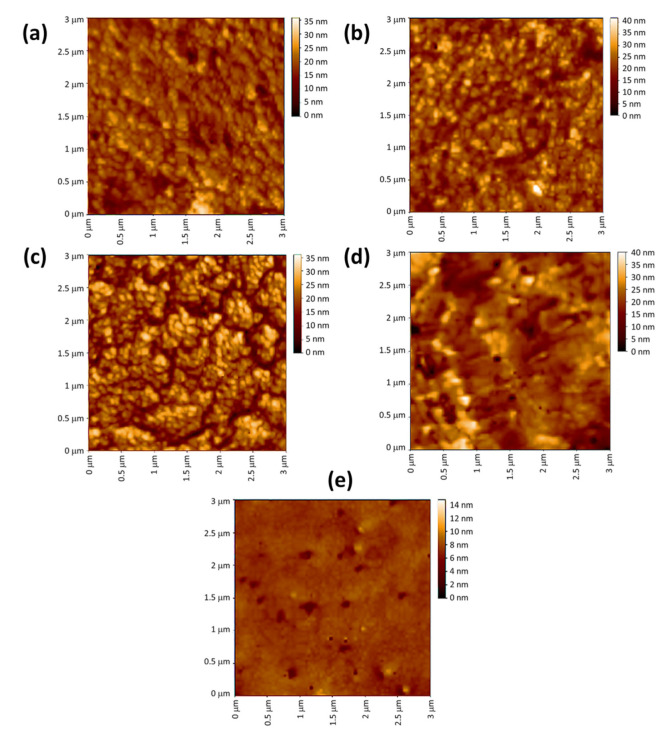
AFM image of (**a**): PVC/L/Ti NPs, (**b**): PVC/L/Cu NPs, (**c**): PVC/L/Co NPs, (**d**): PVC/L/Cr NPs, and (**e**): PVC/L/Ni NPs after irradiation (300 h).

More details about the porosity and roughness of the irradiated PVC surfaces can be obtained using AFM. AFM has the advantage that a vacuum environment is not needed. The roughness in the PVC surfaces after irradiation is due to dehydrochlorination and cleavage of bonds in the polymeric chains [63]. The highest roughness factor (Rq) was found for the irradiated unblended PVC (Figure 10) and the lowest for the Ni NPs blend (Figure 11). The Rq obtained after 300 h of irradiation were 439, 116, 96, 85, 74, and 41 for the PVC (pure), PVC/L, PVC/L/Ti NPs, PVC/L/Cu NPs, PVC/L/Co NPs, PVC/L/Cr NPs, and PVC/L/Ni NPs films, respectively. The Ni NPs led to an improvement in surface roughness by 10.7-fold in comparison to the irradiated pure PVC film. The use of metal oxide NPs in combination with captopril led to an increase in Rq by 5.2–10.3 fold [46]. Modified PVC that contains benzaldehyde, ethylene diamine, and metal oxide NPs led to improvement in the Rq by 4.8–10.9 fold [45]. An additional synthetic step was required, however, to produce the modified polymer. The use of synthesized organotin complexes led to significant improvements in the Rq. The level of improvement in the Rq was found to be highly dependent on the type of organic moiety in the tin complexes [47,48,49,50].

### 3.4. Assessment of PVC Photodegradation by EDX Mapping

**Figure 12:**
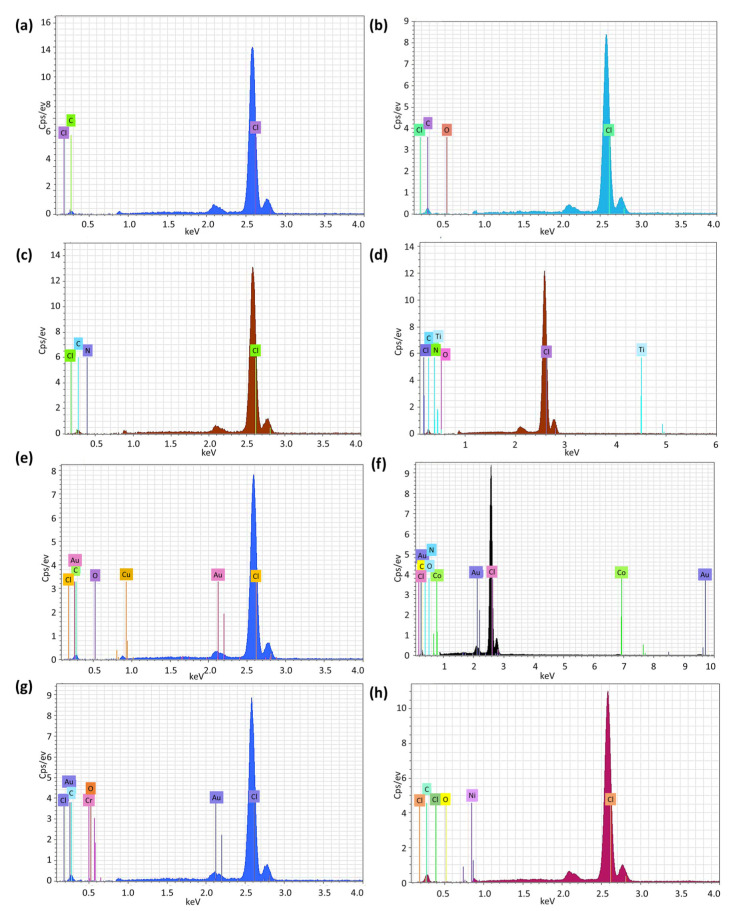
EDX images of PVC films (**a**): pure PVC pre-irradiation, (**b**): pure PVC post-irradiation, (**c**): PVC/L post-irradiation, (**d**): PVC/L/Ti NPs post-irradiation, (**e**): PVC/L/Cu NPs post-irradiation, (**f**): PVC/L/Co NPs post-irradiation, (**g**) PVC/L/Cr NPs post-irradiation, and (**h**): PVC/L/Ni NPs films post-irradiation.

The elemental composition of the non-irradiated PVC blends was analyzed by EDX (Figure 12). It showed all expected elements from the PVC, clotrimazole, and metal oxides present. In addition, EDX was used to examine the role played by metal oxide NPs as photostabilizers. Photodegradation leads to the elimination of HCl and therefore affects the chlorine contents in the PVC blends. Therefore, the chlorine content within the PVC blends was measured before and after irradiation. A low chlorine content indicates high C-Cl bond breaking and therefore a high level of PVC photodegradation.

Following irradiation for 300 h, the percentage of chlorine was found to be lowest for the pure PVC, followed by the blend with just clotrimazole. Blending with metal oxide NPS without exception leads to a much higher chlorine content. The weight percentage of the chlorine for the unirradiated PVC film was ca. 31.5%. The values after 300 h of irradiation were 17.2, 27.2, 30.7, 29.6, 28.3, 30.3, and 31.0 for the PVC (pure), PVC/L, PVC/L/Ti NPs, PVC/L/Cu NPs, PVC/L/Co NPs, PVC/L/Cr NPs, and PVC/L/Ni NPs films, respectively. The results provide clear evidence for the efficiency of the metal oxide NPs in reducing the rate of dehydrochlorination and therefore increasing the resistance of PVC against photodegradation.

Clotrimazole and the metals contribute to stabilization in complementary ways. The polarized bonds linked to the heteroatoms (nitrogen and chlorine) in clotrimazole can coordinate with the polarized C–Cl bonds of the polymer [64]. In addition, the metal oxides acting as basic centers bind to the HCl liberated on UV irradiation [14] producing metal chlorides. The metal oxide NPs used in the current work are efficient UV absorbers and HCl scavengers and therefore increase the resistance of the polymer toward photodegradation.

## 4. Conclusions

A combination of clotrimazole and five metal oxide nanomaterials were mixed in low proportions with poly(vinyl chloride) and thin films were made to assess their abilities to reduce polymeric chains photodegradation. The analysis of infrared spectra and weight loss of the irradiated polymeric blends showed that photodegradation was reduced by the presence of additives and particularly the one containing nickel oxide. Similarly, the assessment of the surface of the irradiated films using different microscopy techniques showed that the damage was much less in the presence of the additives compared with pure poly(vinyl chloride). The additives enhance the photostability of poly(vinyl chloride) by acting as scavengers for hydrochloride and decomposers of peroxides, produced during the photodegradation process, due to the presence of highly basic metal oxides. In addition, the additives act as ultraviolet absorbers since they contain clotrimazole which is aromatic and has heteroatoms. The additives investigated have the advantage that no synthetic steps are needed and no purification is required. A future study is needed to investigate the role played by metal oxides as additives for poly(vinyl chloride) in the absence of any aromatic compounds.

## Data Availability

The data presented in this study are available on request from the corresponding author.
